# Dietary Patterns and Quality of Life in Older Adults: A Systematic Review

**DOI:** 10.3390/nu10080971

**Published:** 2018-07-26

**Authors:** Thara Govindaraju, Berhe W. Sahle, Tracy A. McCaffrey, John J. McNeil, Alice J. Owen

**Affiliations:** 1 Department of Epidemiology and Preventive Medicine, Monash University, Melbourne 3004, Australia; thara.govindaraju@monash.edu (T.G.); berhe.sahle1@gmail.com (B.W.S.); john.mcneil@monash.edu (J.J.M.); 2 Department of Nutrition, Dietetics and Food, Monash University, Clayton 3168, Australia; tracy.mccaffrey@monash.edu

**Keywords:** dietary patterns, quality of life, older adults, systematic review, healthy ageing, nutrition

## Abstract

Dietary patterns may be related to quality of life (QoL) of older adults, although evidence from literature is conflicting. The demographic shifts toward ageing populations in many countries increases the importance of understanding the relationship between diet and QoL in older adults. This review was designed to investigate associations between dietary patterns and QoL in older adults. The systematic review followed the Preferred Reporting Items for Systematic Reviews and Meta-Analyses (PRISMA) guidelines. Eight electronic databases were searched to identify articles published in English from January 1975 to March 2018 that investigated associations between dietary patterns and QoL in older adults. Relevant studies were identified based on set inclusion and exclusion criteria, data were extracted and analysed to examine the relationships and possible implications for public health recommendations. The systematic review included 15 articles (One randomized control trial, six prospective cohorts and eight cross sectional). The studies looked at correlations between different dietary patterns and/or adherence to particular dietary patterns and self-reported QoL or self-rated health status. Excluding two studies which showed no significant association, healthy dietary patterns were associated with better self-rated health and QoL in one or more domains, and adherence to healthy dietary patterns like the Mediterranean diet were significantly associated with improvement in at least one of the QoL domains.

## 1. Introduction

The world population is ageing, and with this, interest in understanding what constitutes healthy ageing is growing. Globally, the population of people aged 60 years and above is expected to double by 2050 reaching 2.1 billion from its current number of 1.4 billion in 2015 [1]. While advances in health care and medicine have increased lifespan, the focus now is on ensuring life years gained are productive and healthy both for older adults and the society in which they live.

Finding ways to improve quality of life as life span increases continues to be a challenge for ageing research. The World Health Organization (WHO) defines quality of life (QoL) as “the individual’s perception of his/her position in life, within the context of culture and value systems in which he/she lives and in relation to his/her objectives, expectations, standards and concerns” [2]. QoL is both subjective and objective in nature, and is often categorized into the five dimensions of physical wellbeing, material wellbeing, social wellbeing, emotional wellbeing, and development and activity [3] and is typical of the living situations of the subjects in question [4]. QoL measures subjective perception of health against the objective assessments of functioning and/or health status, making it unique in a way that two individuals with identical health status can have different QoL, based on their expectation, and resilience towards health or illness, socio economic status, age and social support [5,6,7]. QoL is measured through either generic or idiopathic questionnaires which mainly differ in domains covered, focus on objective as against subjective, self or proxy report and finally the population in question. With use of detailed questionnaires like Short form-12 (SF12) [8] and Short form-36 (SF36) [9], QoL provides a measure of general wellbeing, including both positive and negative features of life, and is an important measure of successful or healthy ageing. Studies have also looked at QoL in the aged population as a means to evaluate health care options especially for subjects with chronic or disabling diseases [10,11]. With increased reliance on patient-reported measures to validate clinical endpoints, QoL serves as an important marker in assessing health status.

QoL in older adults is also likely to be influenced by social aspects including living situations [12], economic dependence [13], age-related physical limitations [14,15] and lifestyle factors including physical activity [16], diet and nutrition [17,18]. Studies have found an Okinawan-based Nordic diet to be associated with better QoL [19]; Red meat, pastries and fast food-based “Western” dietary pattern to have a negative association [20]; and diets like the Mediterranean diet, which have been well researched, to have mixed results [21,22].

Nutrition is an important and readily modifiable risk factor for disease prevention, and studies have consistently shown a relationship between diet and health including in older adults [23,24]. However, diet is a complex construct, and recent research has focused upon dietary patterns as a means to examine the impact of diet on health outcomes at population level [25,26]. Dietary patterns are defined as “the quantities, proportions, variety or combinations of different foods and beverages in diets, and the frequency with which they are habitually consumed” [27], and present the opportunity to account for the complex interactions between foods while measuring the total usual intake of food combinations in individuals and groups [28,29]. With the ongoing development of methods used to assess dietary patterns, including empirical approaches, dietary patterns are now being considered as a basis for dietary guidelines and United States Department of Agriculture recently commissioned a review to understand the evidence for dietary patterns and the associated health outcomes [30]. Traditionally, a priori methods have been used to evaluate dietary patterns. These are indices assessing adherence to established diets based on national guidelines (e.g., Healthy Eating Index) or scientific relevance (e.g., Mediterranean diet). An empirical approach focusing on deriving dietary patterns based on statistical methods like principal component analysis or cluster analysis is another alternative [25]. Both aforementioned approaches have been employed to examine dietary patterns in relation to health outcomes across a range of age groups. Studies have shown adherence to Mediterranean diets to be associated with reduced risk of cardiovascular events [31] and the Dietary Approaches to Stop Hypertension (DASH) diet to influence hypertension and chronic kidney diseases [32,33]. Although dietary patterns identified in each study may be different, some key characteristics of the healthy dietary pattern—high consumption of vegetables, fruits and whole grains, legumes, seafood and low consumption of sweetened foods, refined grains and processed meat—have been proposed to be associated with positive health benefits [34,35]. The role of dietary patterns such as the “Western” diet, characterised by increased consumption of refined foods and saturated fat along with fewer foods from the fruits and vegetable group have also been examined, for healthy factors such as immunity [36], asthma [37] and chronic diseases [38,39,40]. The INTERHEART study examining the relationship between dietary patterns and acute myocardial infarction (AMI) in 52 countries throughout Africa, Asia, Australia, Europe, the Middle East, and North and South America, similarly found “Prudent” diet rich in fruits and vegetables had a protective effect, whereas the highest quartile of “Western” diet characteristics of high fat, salt and meat intake had an adverse effect on AMI across different country settings [41].

Diet intake, and subsequently nutritional status, have been reported to be poor in some older cohorts [42,43,44,45,46,47,48] with migration, living arrangements, loss of loved ones, being unemployed and a lack of social network all leading to changes in diet quality [44,49,50]. Bereavement and a resulting loss of appetite may become more apparent during meal times most likely shared with the lost companion, [51,52] and widowed subjects have been shown to have a lowered quality of diet [52,53]. Dietary adequacy among older adults has been shown to be associated with social network status [54] and financial independence [47,48]. In addition, older adults have been reported to consume less than the recommended intake of fruits and vegetables [55], making them predisposed to the onset of chronic diseases as age advances. It is important to note that dietary patterns in older adults evolve depending on health, psycho-somatic and social conditions, and hence, consideration of these is important to determining the impact of dietary intake on QoL in older adults.

While some studies have examined the association of dietary patterns with hard health outcomes including cardiovascular events, stroke, dementia and mortality, studies examining the associations between dietary patterns and QoL are limited. QoL has mostly been measured in people with diagnosed health conditions [56,57,58,59]; however, examination of this among healthy subjects is increasing. Studying QoL in healthy older adult populations may be especially important as it might have implications on our understanding of healthy ageing, and inform strategies for maintaining health in older years. The limited number of studies that have attempted to measure dietary patterns in relation to QoL have shown that adherence to healthy patterns may have beneficial effects on QoL in women [60] and in older adults [20]. The aim of this review was to evaluate evidence of the association between dietary patterns and QoL in older adults, with the intention of providing a better understanding of the potential for dietary patterns to improve QoL.

## 2. Methods

### 2.1. Search Strategy

Articles were retrieved from eight electronic databases—Medline, Embase, Psychinfo through on the Ovid platform (Ovid technologies Inc., Wolter’s Kluwer, New York, NY, USA) Cochrane through Wiley, Cinhal plus and Ageline through Ebscohost, Web of Sciences and Scopus. Manual searching was done using the “related citations” and bibliography searches of the chosen articles. 

The search used three groups of keywords—“older adults”, dietary patterns” and “quality of life” and their respective synonyms. The search string was developed for Medline through Ovid platform, and this was adapted for the other remaining six databases. Search strategy employed with Medline is attached as Supplement 1. Peer-reviewed journals were searched for articles from January 1975 to March 2018 restricting the search to those published in English, conducted in adults with a mean age of 60 exploring associations between dietary patterns and QoL through validated methods. 

Author T.G. retrieved articles, removed duplicates and screened the articles based on title and abstract and shortlisted articles for full text review with the help of reference management software Endnote (version 8, Clarivate Analytics, Philadelphia, PA, USA). Both Reviewers T.G. and B.W.S. then screened the full text based on eligibility criteria. Both the reviewers extracted data individually and all inconsistencies were verified and resolved by discussion. 

### 2.2. Inclusion and Exclusion Criteria 

All studies which had subjects with the mean age of 60 years, in any setting, were included. Studies done across age groups were considered if stratified results were available for ages 60 and above. Considering that the target population of the review was older adults, “being healthy” and “absence of disease” was difficult to define and hence studies done in both healthy and diseased subjects were included in the review. QoL as measured by any validated measure including but not limited to SF-12, SF-36, World Health Organization Quality of Life (WHOQOL), European Quality of Life scale (EUROQOL)were considered. Dietary intake data collected through food frequency questionnaires and diet history, including 24 h recall methods, both paper-based and online-based were included in the study. Studies that were reviews, case reports or not peer reviewed were excluded from the review.

### 2.3. Data Extraction

Data were extracted in a standard form by both the reviewers independently. The extracts were compared and any difference was verified and resolved through consensus. The extract comprised information regarding general details (title, authors, reference/source, country, and year of publication), study details (name, design, setting, eligibility criteria, sample size and duration) participants (age, gender, ethnicity and health status), and results (type of analysis, outcome measures, time points of data collection, primary results, author conclusions).

### 2.4. Overall Quality of Studies 

Quality assessment of the studies included in the review was performed using Effective Public Health Practice project (EPHPP) developed “Quality Assessment Tool for Quantitative Studies”. The tool has been validated [61] and compared to other instruments and has been found to be reliable and consistent [62]. The tool assesses the studies based on six components—selection bias, study design, confounder, blinding, data collection methods and withdrawals and dropouts. The individual components were rated as strong, moderate or weak based on the quality assessment tool for quantitative studies dictionary and a final global rating for the paper is given based on the individual component ratings. The paper was rated strong if there were no weak ratings, rated moderate if there was one weak rating and rated weak if there was more than one weak rating. Both reviewers rated the study independently and discussed the ratings. All differences were discussed and an agreement was reached.

## 3. Results

**Figure 1:**
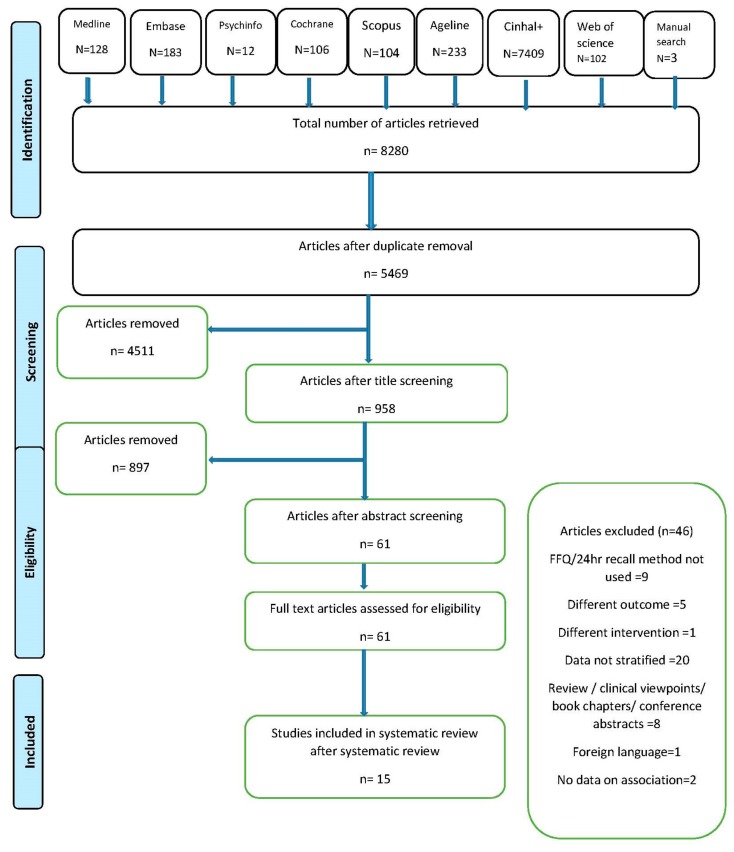
Preferred Reporting Items for Systematic Reviews and Meta-Analyses (PRISMA) flow diagram of identification, screening and selection process for included articles.

The review followed PRISMA guidelines for systematic review reporting [63] and was registered with PROSPERO, international prospective register of systematic reviews (CRD42017068407). Figure 1 depicts the PRISMA flowchart [64] for the study. A total of 8280 articles from eight electronic databases and a further three from manual searching were retrieved. About 2811 duplicates were removed and the remaining articles were screened based on the title. A total of 958 articles were found to be eligible for abstract screening and 61 articles qualified for the full text review. Forty-six articles had to be excluded from the review for not meeting the inclusion—exclusion criteria of which 20 had results that were not and could not be age stratified, nine did not use dietary measures defined in inclusion criteria, five recorded different outcomes, one used dietary pattern as a component of larger intervention, one did not investigate association with outcome, one was published in foreign language and the rest were in the form of clinical views, conference papers and reviews and were thus excluded. Efforts were made to contact authors of eligible studies without stratified results, but were unsuccessful in procuring data for the target age group. A total of 15 articles were included in the final review. 

There were a total of six prospective cohort studies, eight cross-sectional studies and one randomised controlled trial (RCT) identified. Table 1 describes the characteristics of the studies included in the review. Papers where cross-sectional data were derived from longitudinal studies were considered cross sectional. Seven of the studies were conducted in European countries; four in USA; one multicentre study with participants in USA, UK and Canada; one in Hong Kong; and two in Australia. The RCT [65] with 48 participants had the lowest sample size, six studies had a sample size of <1000, four studies had sample size between 1000 and 3000 and the rest had a sample size of >3000 subjects.

The studies measured the association of dietary patterns derived from a priori dietary indexing methods or empirical approaches or both with QoL in older adults. While nine studies [21,66,68,71,73,74,75,76,77] measured baseline association between dietary patterns and QoL, four studies measured diet at baseline and QoL at both baseline and/or follow up [22,67,70,72], one study measured both diet and QoL at multiple points in the study [69] and one study measured the association of change in dietary patterns made in relation to a chronic disease diagnosis and its impact on QoL [72]

Most studies measured QoL using standard questionnaire-based measures of SF-12 or the longer version SF-36, with two studies [67,77] using the self-rated health status. Studies using SF-12 or SF-36, also reported physical composite scores (PCS) and mental composite scores (MCS) along with mean scores for eight identified domains. Two studies used validated Spanish versions of the SF-12 and SF-36 [22,74], one used Health and Activity Limitation Index (HALEX) [76], and one used RAND 36. Some studies used disease-specific QoL questionnaires including the European Organisation for Research and Treatment of Cancer—Quality of Life questionnaire (EORTC-QLQ-C30) [68], Audit of Diabetes Dependent Quality of Life (ADDQOL-19) [73] and Minnesota Living with Heart Failure Questionnaire [65]. One study used SF-36 alongside Functional Assessment of Cancer Therapy-Colorectal (FACT-C) [69]. 

Dietary assessment methodology also varied between studies with 10 studies using food frequency questionnaires (FFQs) of varying versions, two studies measuring modified versions of diet history [67,72], one measuring 24 h recall [75], one a dietary screening tool (DST) [76], and one assessing multiple measures of diet including a computerised diet history and a FFQ [22].

Almost all studies used dietary indices to derive dietary patterns with only one study deriving patterns using statistical approaches [77]. Indices used varied from those developed to measure adherence to Mediterranean diet, national or international guidelines, food variety and diversity and compliance to specific diets such as DASH with three studies using multiple indices [22,70,74]. The indices used included Diet Quality Index (DQI) [74] and DQI-International [66], Healthy Eating Index (HEI05) [75,76], DASH diet index score [65], with some studies [22,68] developing their own unique indices

Of the studies that measured adherence to Mediterranean diet, four [22,67,70,71] used Mediterranean diet score (MDS) by Trichopoulou et al. [78] or modified versions thereof one [22] used PREDIMED score [79], one [21] used adherence to Mediterranean diet (aMED) [80] and one [73] used relative Mediterranean diet score (rMED) [81].Of the studies that measured adherence to dietary guidelines, two [69,70] were based on Australian national guidelines [82] and one [65] on the DASH diet index score [83]

Most studies included in the review were rated as moderate quality studies with one study rated as weak and four studies rated as high quality studies, mostly based on study design and drop outs. All cross-sectional studies scored lower for study design, based on the tool guideline. Studies included validated measures of diet and QoL and this information was available for most studies in the article, or in the cited literature. Almost all studies reported a number of withdrawal and dropout rates. The reviewer ratings for each of the studies is attached as supplement 2.

In summary, 13 out of the 15 studies included in the review found dietary patterns are associated with QoL in older adults. The two studies that did not find any association with QoL were both longitudinal studies conducted in older Europeans with existing comorbidities and measured health status in relation to a high quality diet like the Mediterranean diet, and were not dissimilar to the studies with significant association [22,67]. All the studies specifically involving subjects with chronic diseases or at risk of chronic diseases found higher QoL scores with better diet quality [65,68,72,73,75]. The heterogeneity in assessment methods used and study designs did not allow the data to be synthesised by meta-analysis. 

## 4. Discussion

This review supports the existence of an association between dietary patterns influence and QoL in older adults. The majority of the studies (87%) included in this review found that subjects with higher diet quality had higher mean scores on QoL scale, with the exception of two studies (13%) reporting no association. Most of the studies have been conducted in developed countries and in older adults with existing co-morbidities. Although the results have been consistent across studies using a variety of validated instruments to assess diet and QoL, the lack of randomised trials in a healthy older adult population mandates exercising caution in generalising these results, as reverse causation cannot be excluded.

Of the studies reporting positive association, higher quality diets were shown to be associated with better QoL in five studies [65,66,70,71,74] and greater adherence to Mediterranean diet in three [21,73,76]. Gopinath et al. [69] found that among older adults with higher baseline diet scores, greater QoL was seen across three domains prospectively over 5 years. Another study [71] prospectively examining the relationship between dietary patterns through multiple indices found positive associations between diet quality and five domains of QoL and PCS when using dietary guideline index (DGI), and four domains of QoL with Recommended Food Score (RFS). Apart from this, associations were also found between adherence measured through MDS and general health and vitality domains.

With increasing interest in a ’whole diet’ approach to understanding the relationship between diet and health, reviews have investigated associations between dietary patterns and depression [84], colorectal cancer [85], breast cancer risk [86] and other outcomes including nutrient adequacy, biological outcomes, morbidity and mortality [87]. There is a paucity of studies examining the association between dietary patterns and QoL in older adults. One other detailed review [88], although not entirely focused on QoL and including studies with slightly younger cohorts (>45 years), has looked at the impact of dietary patterns on QoL as a component of ‘successful aging’. That review found the majority of studies reported an association between a healthier dietary pattern and better health outcomes, but the review herein has been the first to our knowledge to examine the relationships between dietary patterns on QoL in older adults.

Subjects with intermediate cardiovascular risk (defined as 5–15% 10-year risk of developing cardiovascular disease) showed mental health scores positively associated with diet quality and higher scores on Mental Composite Scores (MCS), social functioning and vitality with greater adherence to Mediterranean diet [74]. A study by Lewis et al. [72] measuring the impact of dietary changes post-cancer diagnosis, found that subjects who made healthful dietary changes had significant improvements in PCS and functional wellbeing. In older adults with heart failure, adherence to the DASH diet was found to be associated with improved QoL scores at 3 months [65]. Samieri et al. [77] derived sex-specific dietary patterns using statistical approaches and reported that men in the “pasta eaters” cluster and women in “ biscuits and snacking “ cluster were more likely to report poor perceived health. In that study, five different dietary patterns were derived for men and women each using hybrid clustering methods, the clusters being based on average weekly servings. Two studies examined diet as a component of lifestyle score, and one [75] found that a lifestyle score including BMI < 30 kg/m^2^, healthy diet, moderate recreational activities and non-smoking status was related to gHRQoL among colorectal cancer survivors. This finding was supported by a study which found that health status was associated with both physical activity and non-smoking status, even when diet was not [67].These results might indicate a role for overall lifestyle modification in improving the QoL, as opposed to the focus on diet alone. While studies have investigated the role of lifestyle factors including diet, physical activity, and smoking among others, such studies were excluded owing to heterogeneity and complexity of the assessments involved in those studies. The authors acknowledge that the combined effect of lifestyle factors is an interesting area worthy of further research.

Among the 13% of studies contributing to the present review that found no association between diet and QoL, Perez-Tasigchana et al. [22] used multiple indices to measure adherence and found that while PREDIMED scores were linked to a slightly better PCS, there was no relationship between Mediterranean Diet Score and any of the QoL domains. The studies were conducted 10 years apart and both the cohorts used different instruments to measure diet and QoL, and obtained consistent results. Haveman-Nies et al. [67] in their longitudinal study, used a modified Mediterranean score and measured self-rated health and noted that having a high quality diet did not delay the deterioration in self-rated health status as measured through baseline, mid-point and end of follow up.

Despite growing interest in dietary patterns and their measurement through empirical approaches, only one study used cluster analysis to derive dietary patterns making dietary indices still the popular measure to derive diet quality. The scoring methodology and the data used to generate the score differ from study to study, for the lack of standardised procedures. Guideline-based indices like DQIs are mostly country-specific and are not valid for all countries. The components vary across indices with HEI [89] comprising of nine adequacy components and three moderation components, DQI [90] and DQI-I [91], both comprising of four components—adequacy, variety, moderation and overall balance, with scores based on grams/day, frequency or number of servings or daily allowances. Further research, especially from longitudinal studies with older adults on diet and QoL, is needed. Studies with dietary patterns derived from statistical approaches, might help address issues related to guideline-based dietary indices. Empirical approaches, though they have their limitations, might be better in identifying relatable food groups or similar populations. Mediterranean diets were still the most commonly studied diet pattern in our review, followed by diets based on national guidelines with one study assessing disease-based diet compliance with the DASH diet. 

### Strengths and Limitations of This Study

This review is a broad analysis of association between dietary patterns and QoL in older adults, and included studies from 1975, covering even the initial publications concerning “dietary patterns”. The protocol of this review was registered in PROSPERO and followed the Preferred Reporting Items for Systematic Reviews and Meta-Analyses guidelines. The methodological quality of studies was assessed by Effective Public Health Practice Project (EPHPP) tool and we anticipate that the results of the review can inform public health practice and policies regarding diet in older adults for a better QoL. 

The review combines both healthy and diseased populations for the lack of data in healthy older adults. This may limit extrapolation to the entire older adult population, although we believe that results will not be too dissimilar, considering co-morbidities are common in this age group. The study included articles published in English only and hence could have lost research of consequence published in other languages. The studies included in the review are largely observational studies, with more than half the studies being cross sectional and cannot be used to imply causality. Lastly, the review might be liable to the inherent bias that is associated with self-reported measures of diet and QoL, in addition to well described random and systemic errors in epidemiological-scale dietary assessment [92].

## 5. Recommendations

Studies looking at the relationship between QoL and dietary patterns in older adults are few and lacking in quality. Results from large, well conducted longitudinal studies are required to better understand the relationship between dietary patterns and QoL among older adults. Also lacking are the sensitive tools to measure diet and QoL, and their respective validations in older adult populations. Very few studies use FFQs and diet diaries specifically validated in elderly populations [93], mostly relying upon validations done in general populations [94]. When dietary assessment tools are validated among older adults, they are thought to produce relatively accurate results [95,96] although they may underestimate intake [97]; however, questions remain regarding the participant burden from these tools in populations which may be experiencing diminishing cognitive and physical abilities. The most popular tools that measure QoL make no effort to capture the dietary behaviours, and this needs to be considered for future research [98,99]. Considering the growing proportion of older people in many countries around the globe, and the imperative to maintain QoL in older age, developing and validating tools that can accurately measure QoL and its determinants, including diet, are of great public health relevance.

## Figures and Tables

**Table 1:** Characteristics of the studies included in the systematic review.

Study and Setting	Study Design	Sample Size	Mean Age ^1^	(A)Dietary Intake Assessment (B) Pattern Analysis Method (C) Adjustment Variables	QoL Measure	Results	Study Quality ^2^
Woo, J. et al. [66] Hong Kong	Cross sectional	3378	72.5 ± 5.2	(A):FFQ (B): DQI-I with 4 aspects—variety, adequacy, moderation and overall balance: Range 0–94, high scores better quality (C): Age, sex, socioeconomic status and districts	SF-12	Better dietary quality is associated with better self-rated physical and mental health. PCS: β = 0.0689 (*p* < 0.0001) MCS: β = 0.0693 (*p* < 0.0001)	moderate
Haveman-Nies et al., SENECA [67] Europe (Belgium, Denmark, Italy, The Netherlands, Portugal, Spain and Switzerland)	Longitudinal	480	72.6 ± 1.6	(A) Diet history (B) Modified Mediterranean score: Range 0–7, high scores indicate better quality. (C): Age, country	Self-rated health status	No association observed between diet quality and risk of deterioration of health status. Risk of deterioration in health status resulting from low dietary quality: OR (95% CI): Men 1.1 (0.5, 2.3); women 1.4 (0.7, 2.7)	weak
Schlesinger et al. [68] Northern Germany	Cross sectional	1389	69 (64–73)	(A): 112 item web-based FFQ/ (B): Dietary Index: Two indices namely ’favorable‘ and ’unfavorable‘ were generated (C): Age sex and SES	EORTC QLQ-C30	Those with a favorable’ diet had a reduced odds of having a low gHRQoL; OR (95% CI): 0.79 (0.63–0.99)	moderate
Perez-Tasigchana et al., UAM-cohort, & Seniors-ENRICA [22] Spain	Longitudinal	2376	≥60	(A):14 item paper-based FFQ (B): Mediterranean dietary pattern index (UAM-MDP): Range −2 to 6, low score indicates less healthy diet. (C): Age, sex, level of education, smoking, BMI, physical activity, comorbidities	SF-36 (Spanish version)	No significant association between UAM-MDP and PCS and MCS.	strong
		1911	≥60	(A): Dietary history-Enrica (B): PREDIMED score: Range 0–14 high scores better adherence MDS :Range 0–9 high scores better adherence (C): Age, sex, level of education, smoking, BMI, physical activity, comorbidities	SF-12	Higher PREDIMED score was associated with slightly better PCS score. Compared to those in the lowest tertile, PCS: b = 0.55 (−0.48 to 1.59) for tertile 2, 1.34 (0.21 to 2.47) for tertile 3PREDIMED score not significantly associated with a better MCS score. [/MCS: tertile 2, b = −0.25 (−1.31 to 0.80) and tertile 3, b = 0.56 (−0.58 to 1.71)]	
						MDS not associated with PCS or MCS	
Gopinath et al., BMES [69] Australia	Longitudinal	895	67.1 ± 7.4	(A): 145 item FFQ (B): Total Diet Score to assess adherence to dietary guidelines for Australian adults: Range 0–20, higher scores better adherence (C): Age, sex, receipt of pension payment, home ownership, admission to hospitals, walking disability, living alone, 5 or more co morbidities, cognitive and visual impairment	SF-36 and FACT-C	Adherence to dietary guidelines at baseline was associated with significantly better QoL in four domains after 5 years. Participants in the highest vs. lowest quartile of baseline total diet scores had adjusted mean scores 5.6, 4.0, 5.3, and 2.6 units higher in these SF36 domains 5 years later	moderate
Milte et al., WELL [70] Australia	Longitudinal	2457	59.9 (65–55)	(A): 111 item FFQ (B): DGI: Range 0–130 higher scores reflect greater compliance with dietary guidelines RFS: Range 0–49, with higher scores associated with greater diet quality MDS: Range 0–8, higher scores reflecting greater adherence to Mediterranean diet. (C): Model 1: adjusted for age, sex, education and urban/rural location. Model 2: additionally adjusted for smoking and total physical activity. Model 3: additionally adjusted for BMI	RAND 36	Older adults with better quality diets report better health-related QoL, with additional associations with emotional wellbeing observed in women. Better diet quality by DGI was associated with better self-reported HRQoL on the physical function (OR = 1.56, 95% confidence intervals (CI): 1.22–1.99), bodily pain (OR = 1.29, CI: 1.01, 1.63), general health (OR = 1.72, CI: 1.36, 2.19), energy (OR = 1.51, CI: 1.19, 1.92), emotional wellbeing (OR = 1.36, CI: 1.08, 1.72) and PCS (OR = 1.46, CI: 1.15, 1.86).	strong
						A higher RFS was associated with better HRQoL on the physical function (OR = 1.43, CI: 1.13–1.82), general health (OR = 1.41, CI: 1.12, 1.78), energy (OR = 1.55, CI: 1.22, 1.96) and emotional wellbeing (OR = 1.41, CI: 1.12, 1.77)	
						MDS score in the top quartile was associated with a better score on the energy scale (OR = 1.53, CI: 1.11, 2.10). An association between MDS and general health was also observed after adjustment for smoking and physical activity (OR = 1.52, CI: 1.11, 2.08)	
Zaragoza-marti et al. [71] Spain	Cross sectional	351	71.06	(A): MEDIS-FFQ (B): MDS: Range 0–9, higher the score higher the adherence (C): Age, hours of physical activity, educational level, BMI, blood cholesterol, blood glucose levels and blood pressure	SF 12	Adherence to MD is positively related to both PCS and MCS of SF12 for both sexes. Regression coefficients for the relationship between Mediterranean diet score with women (MCS (0.07, CI:(−0.96–0.23, *p* < 0.001) and PCS (0.19, CI (0.04–0.34, *p* = 0.020)) and men (MCS (0.01, CI: −0.12–0.29, *p* = 0.004 and PCS (0.05, CI: 0.17–0.20, *p* = 0.060))	moderate
Veronese et al., Osteoarthritis Initiative [21] United States	Cross sectional (sub study of a large longitudinal study)	4470	61.3 ± 9.2	(A): 77 item Block brief 2000 FFQ (B): Modified MDS: Range: 0–55 high scores better adherence (C): Age, sex, race, BMI, education, smoking status, total energy intake, Charlson co morbidity index, use of analgesic drugs, annual income	SF-12	Higher adherence to med diet is associated with better QOL. Those with higher aMED showed significantly higher PCS (quintile 5: 50 ± 8.5 compared to quintile 1: 47.2 ± 9.8; *p* < 0.0001) and MCS (quintile 5: 54.5 ± 7.6 compared to quintile 1: 53.2 ± 8.8; *p* < 0.0001)	moderate
Lewis et al. [72] United States	Longitudinal	265	64.5 ± 10.3 (Caucasians)/60.7 ± 10.2 (African American)	(A): 44 item Diet history questionnaire (B): Dietary index scored from −30 to 30 Higher scores better adherence. (C): Age, sex, follow up time, education, Socio economic factors, BMI, alcohol consumption, smoking and physical activity	SF-12	Subjects who improved dietary quality exhibited positive changes in QOL-significant changes observed in functional wellbeing (0.14, CI: 0.05–0.07, *p* ≤ 0.01), functional assessment of cancer therapy-general total score (0.19, CI: 0.01, 0.37, *p* = 0.04) and Physical composite score of SF12 (0.23,CI: 0.05–0.41, *p* = 0.01)	strong
Alcubierre et al. [73] Spain	Cross sectional	294 (146 DR and 148 NDR)	No Diabetic retinopathy: 57.9 ± 10.3	(A) Semi quantitative FFQ 101 items (B): rMED: Range, 0–18 Adherence was rated as low (0–6), medium (7–10) and high (11–18) (C):Adjustments varied for different components of the QoL domain and included age, ethnicity, insulin treatment, retinopathy, diabetes duration another diabetes related factors	ADDQOL-19	rMED was significantly associated with HRQOL dimensions of travels, self-confidence, freedom to eat and freedom to drink. rMED > 8, positively associated with self confidence (*p* = 0.015), freedom to eat 0.839 (*p* = 0.037) and freedom to drink 1.150 (*p* = 0.015)	moderate
			Diabetic retinopathy: 60.5 ± 8.8				
Rifai et al. [65] United States	Randomised controlled trial	48	DASH group (60) and comparison group (64)	(A): FFQ/food diaries (B): DASH diet index: Range 0 to 11, with higher scores indicating higher levels of concordance. (C): N/A	MLHF	Adhering to the DASH diet improved QoL scores at 3 months; improved MLHFQ scores at 3-month follow-up (21 vs. 39; *p* = 0.006)	strong
Sanchez-Aguadero et al. MARK study [74] Spain	Longitudinal	314	61.1 ± 8.4 (35–74)	(A): FFQ with 18 food groups divided into three categories (B): DQI: Range 18–54, with higher scores associated with better diet quality. aMED: Score ≥ 5 meaning good compliance (C): Age, sex, hypertension, dyslipidaemia and Charlson Comorbidity Index	Spanish version of the SF-12 v.2	In those at intermediate cardiovascular risk, DQI was directly related to the mental component score (r = 0.127, *p* < 0.05) and mental health (r = 0.121, *p* < 0.05), in bivariate analyses	moderate
						Greater adherence to the Mediterranean diet was associated with higher scores on the SF-12 mental component, social functioning and vitality and DQI showed an association with the mental component score. Bivariate correlation: The Mediterranean Diet (total score) was related to the mental component (r = 0.164, *p* < 0.01) as well as social functioning (r = 0.172, *p* < 0.01) and vitality (r = 0.122, *p* < 0.05). Multiple linear regression: 1.177 point increase in the mental component for each increase of 1 point in the Mediterranean diet adherence score (*p* < 0.01), Vitality (β = 0.958 and 0.990) and Social Functioning (*p* < 0.05 and *p* < 0.01) domains maintained association post adjustments.	
Mosher et al., RENEW [75] United States, UK and Canada	Cross sectional	641	73 ± 5	(A): 24 h dietary recalls (B): HEI05: Range 0–100 with scores above 80 indicating good diet quality. (C): age, race, level of education, and number of comorbidities	SF-36	Diet quality was positively associated with physical functioning (β = 0.10, *P*_s_ < 0.005) and vitality (β = 0.095, *P*_s_ = 0.01)	moderate
Ford et al., GRAS [76] United States	Cross sectional	4009	Males: 81.3 ± 4.2, Females: 81.5 ± 4.5	(A): DST (B): HEI 05: Range :0–100 (<60 considered “unhealthy”, 60–75 “borderline”, and >75 “healthy”) (C): BMI, disease burden, sex, education, age, smoking status, living situation and self-vs. proxy report	HALex	Poor diet quality, as assessed by the DST, is associated with lower HRQoL. HALex scores were significantly lower for participants with dietary intakes categorized as unhealthy (<60) (0.70, 95% CI 0.69, 0.72, *p* < 0.05) or borderline (60–75) (0.71, 95% CI 0.70, 0.73, *p* < 0.05) compared to those scoring in the healthy range (>75) (0.75, 95% CI 0.73, 0.77).	moderate
Sameiri et al., Three City Study [77] France	Cross-sectional	1724	76.0 ± 4.9	(A): 148 item FFQ (B): Mixed method combining hybrid clusters to derive sex-specific dietary patterns. Five dietary patterns were identified in men and women each. (C): Sociodemographic variables, and comorbidities	Self-rated health status	Men in the “pasta eaters” cluster had greater risk of reporting poor health (odds ratio [OR] 1.91; 95% CI, 1.21–3.01) than the “healthy” cluster. Women in the “biscuits and snacking” cluster (*n* = 162; 15%) had greater risk of poor perceived health (OR 1.69; 95% CI, 1.15–2.48) compared to “healthy” eaters.	moderate

^1^ Age given as Mean ± SD or mean (range) or minimum age (≥) in years; ^2^ Quality of studies as assessed by Effective Public health Practice Project (EPHPP) quality assessment tool. *SENECA* Survey in Europe on Nutrition and the Elderly; a Concerted Action; *Seniors—ENRICA* Study on Nutrition and Cardiovascular Risk in Spain; *UAM* Universidad Autonoma de Madrid; *BMES* Blue Mountain Eye Study; *MARK* Improving interMediAte Risk management; *WELL* Wellbeing, Eating and exercise for a Long Life; *RENEW* Reach—out to Enhance Wellness trial; *GRAS* Geisinger Rural Aging Study. *HRQol* Health related quality of life; *EORTC QLQ-C30* European Organisation for Research and Treatment of Cancer, quality of life core questionnaire; *FACT-C* Functional assessment of cancer therapy-colorectal; *gHRQol* Global Health related quality of life; *SF12* Short form survey 12; *SF36* Short form survey 36; *Rand-36* Rand 36 item health survey; *ADDQOL*-Audit of Diabetes dependent quality of life; *MLHF* Minnesota Living with Heart Failure Questionnaire; *HALeX* Health and activities limitation index. *FFQ* Food Frequency Questionnaire; *DASH* Dietary Approaches to Stop Hypertension; *rMED* Relative Mediterranean diet score; MDS Mediterranean diet score; *MD* Mediterranean diet; *PREDIMED* Prevención con Dieta Mediterránea; *RFS* Recommended Food score; *DGI* dietary guideline Index; *DST* Dietary Screening Tool; *DQI* Diet quality index ; *aMED* Adherence to Mediterranean diet ; *PCS* Physical component score; *MCS* Mental component score.

## Supplementary Materials

The following are available online at http://www.mdpi.com/2072-6643/10/8/971/s1, Table S1: Search Strategy, Table S2: Quality of Studies.
