# Supplementary material for: Dietary Patterns and Quality of Life in Older Adults: A Systematic Review
Source: Nutrients. 2018 Jul 26;10(8):971. doi: 10.3390/nu10080971 (PMC6115962; doi:10.3390/nu10080971)
Supplement: Supplementary file 1 [file nutrients-10-00971-s001.zip › Supplement 2 _Quality of studies.docx]

| Study reference, Name | Selection Bias | Study design | Confounders | Blinding | Data collection Method | Withdrawals and dropouts | Global rating^ |
| --- | --- | --- | --- | --- | --- | --- | --- |
| Woo J et. Al,[66] | 2 | 3 | 1 | 2 | 1 | 1 | 2 |
| Haveman-Nies et. Al, SENECA[67] | 3 | 2 | 2 | 2 | 1 | 3 | 3 |
| Schlesinger et. Al,[68] | 2 | 3 | 1 | 2 | 1 | 1 | 2 |
| Perez- Tasigchana et, al, UAM-cohort, & Seniors- ENRICA [22] | 1 | 2 | 1 | 1 | 1 | 1 | 1 |
| Gopinath et al, BMES[69] | 1 | 2 | 1 | 2 | 1 | 3 | 2 |
| Milte et al, WELL[70] | 2 | 2 | 1 | 2 | 1 | 1 | 1 |
| Zaragoza -marti et al, [71] | 2 | 3 | 1 | 2 | 1 | 2 | 2 |
| Veronese et al, Osteo arthritis initiative[21] | 1 | 3 | 1 | 2 | 1 | 2 | 2 |
| Lewis et al,[72] | 2 | 2 | 1 | 2 | 1 | 2 | 1 |
| Alcubierre et al,[73] | 2 | 3 | 1 | 2 | 1 | 2 | 2 |
| Rifai et al,[65] | 1 | 1 | 1 | 2 | 1 | 1 | 1 |
| Sanchez- aguadero et al, MARK  study[74] | 2 | 3 | 1 | 2 | 1 | 2 | 2 |
| Mosher et al, RENEW[75] | 1 | 3 | 1 | 2 | 1 | 2 | 2 |
| Ford et al, GRAS[76] | 1 | 3 | 1 | 2 | 1 | 2 | 2 |
| Sameiri et al, Three city study[77] | 2 | 3 | 1 | 2 | 1 | 2 | 2 |

^Global Rating: Overall rating based on component scores: 1- Strong, 2-Moderate, 3-Weak
