# Supplementary material for: Dietary Patterns and Quality of Life in Older Adults: A Systematic Review
Source: Nutrients. 2018 Jul 26;10(8):971. doi: 10.3390/nu10080971 (PMC6115962; doi:10.3390/nu10080971)
Supplement: Supplementary file 1 [file nutrients-10-00971-s001.zip › Supplement 1_search strategy.docx]

| # | Search terms | Results |
| --- | --- | --- |
| 1 | "Quality of Life"/ | 153455 |
| 2 | QoL.mp. | 24145 |
| 3 | hrQoL.mp. | 9950 |
| 4 | Health Status/ | 71345 |
| 5 | 1 or 2 or 3 or 4 | 212703 |
| 6 | elderly.mp. | 195950 |
| 7 | Aged/ | 2705522 |
| 8 | "Aged, 80 and over"/ | 763647 |
| 9 | older.mp. | 301930 |
| 10 | 6 or 7 or 8 or 9 | 2896841 |
| 11 | Dietary pattern.mp. | 2157 |
| 12 | Diet score.mp. | 479 |
| 13 | Diet, Mediterranean/ or Diet, Vegan/ or Diet, Western/ or Diet, Vegetarian/ or Healthy Diet/ | 5669 |
| 14 | (Diet adj1 quality).mp. [mp=title, abstract, original title, name of substance word, subject heading word, keyword heading word, protocol supplementary concept word, rare disease supplementary concept word, unique identifier, synonyms] | 1995 |
| 15 | FODMAPS.mp. | 81 |
| 16 | ((Diet or eating) adj index).mp. [mp=title, abstract, original title, name of substance word, subject heading word, keyword heading word, protocol supplementary concept word, rare disease supplementary concept word, unique identifier, synonyms] | 714 |
| 17 | 11 or 12 or 13 or 14 or 15 or 16 | 9622 |
| 18 | 5 and 10 and 17 | 139 |
| 19 | limit 18 to (english language and yr="1975 -Current") | 128 |
